# Continuous decline in bone mineral density and deterioration of bone microarchitecture 7 years after Roux-en-Y gastric bypass surgery

**DOI:** 10.1530/EJE-19-0741

**Published:** 2020-01-14

**Authors:** Stinus Hansen, Niklas Rye Jørgensen, Anne Pernille Hermann, Rene Klinkby Støving

**Affiliations:** 1 Department of Medicine, Hospital South West Jutland, Esbjerg, Denmark; 2 Institute of Regional Health Research, University of Southern Denmark, Odense, Denmark; 3 Department of Clinical Biochemistry, Rigshospitalet, Glostrup, Denmark; 4 OPEN, Odense Patient data Explorative Network, Odense University Hospital/Institute of Clinical Research, University of Southern Denmark, Odense, Denmark; 5 Department of Endocrinology, Odense University Hospital, Odense, Denmark; 6 Institute of Clinical Research, University of Southern Denmark, Odense, Denmark

## Abstract

**Objective:**

Roux-en-Y-gastric bypass (RYGB) surgery is an effective treatment for morbid obesity. A possible overlooked side effect is negative bone metabolic consequences.

**Design:**

A seven-year prospective study following ten women and seven men after RYGB (baseline mean age 43 ± 8 years, BMI 42 ± 6 kg/m^2^).

**Methods:**

Lumbar spine and total hip bone mineral density (BMD) using dual energy x-ray absorptiometry, distal radius and tibia bone geometry, volumetric BMD, microarchitecture and finite element estimated bone strength using high-resolution peripheral quantitative CT and biochemical markers of bone remodelling were assessed at baseline, 2 and 7 years.

**Results:**

Compared to baseline, body weight was 24 ± 10% lower after 2 years and 21 ± 11% after 7 years. During the 7 years of follow-up, radius and tibia vBMD had declined 13 ± 8% and 8 ± 7% from baseline to 2 years and further 10 ± 7% and 7 ± 8% from 2 to 7 years (all *P* < 0.001). At both radius and tibia, cortical thickness declined and cortical porosity increased. From baseline to 7 years, there were clear indications of deteriorations of the trabecular network with fewer, more widely spaced and more in-homogeneously distributed trabeculae in both radius and tibia. Overall, declines in estimated bone strength of 16 ± 9% in radius and 16 ± 7% in tibia were observed (both *P* < 0.001).

**Conclusion:**

Seven years after RYGB, evidence of continuous declines in BMD and ongoing deterioration of bone microarchitecture and reduced estimated bone strength compared to baseline and 2 years post-surgery results were found. These findings emphasize the need for regular assessment of bone health in patients with prior RYGB.

## Introduction

In individuals with morbid obesity, bariatric surgery causes sustained weight reduction, induces remission or improvement in obesity-related comorbidity and lower mortality rates (1). In recent years it has become clear, however, that bariatric surgery and malabsorptive procedures such as Roux-en-Y-gastric bypass, in particular, may have adverse effects in the skeleton (2). Epidemiological studies have documented that following RYGB the risk of fracture increases with indications that this risk may further increase with time from surgery (3, 4). 

A number of studies have documented substantial declines in bone mineral density (BMD) in the initial year after bariatric surgery. The decline in proximal femur BMD is particularly pronounced and correlates with the magnitude of weight loss suggesting that this at least, in part, reflects the relative unloading of the skeleton (5). Skeletal homeostasis is nevertheless affected even after weight loss ceases which typically occurs approximately 12 months after surgery. Biochemical markers of bone remodelling rise rapidly following surgery and then gradually decline although they typically plateau above pre-surgery levels (6). The pathophysiology is thought to be multifaceted and may include lower gastrointestinal absorption of calcium and vitamin D causing secondary hyperparathyroidism (7), hormonal changes including alterations in the secretion of adipokines and gastrointestinal peptides with effects on bone remodelling cells as well as changes in the amount of bone marrow fat (2).

In the first to second year after RYGB, bone loss typically decelerates although BMD continues to decline at both the lumbar spine and proximal femur (2). Although assessment of BMD using dual-energy x-ray absorptiometry (DEXA) may be imprecise in settings with large changes in body composition, similar declines have been shown using central quantitative CT (8). Bone is also lost at the peripheral skeleton, and studies using high-resolution peripheral quantitative CT (HR-pQCT) have reported changes in both cortical and trabecular microarchitecture with corresponding declines in estimated bone strength in the initial years after RYGB (8, 9). 

Only few studies have examined bone changes beyond the first years after RYGB. A prospective cohort study found continuous declines in both spine and hip BMD 5 years after RYGB (10). Similarly, a recent prospective study with 5 years of follow-up reported declines in BMD at the lumbar spine and proximal femur and negative changes in bone microarchitecture and estimated bone strength in radius and tibia (11). 

These short and intermediate term studies suggest ongoing bone loss after RYGB, although yet there are no studies that have prospectively assessed skeletal changes beyond 5 years of surgery. Therefore, the aim of this study was to examine BMD, bone microarchitecture and estimated bone strength and biochemical markers of bone remodelling 7 years after RYGB. Based on previous evidence, we hypothesized that RYGB induces an ongoing increase in bone remodelling with declining BMD and deficits in bone microarchitecture and estimated bone strength. 

## Subjects and methods

This is a prospective cohort study, and details regarding recruitment and 1- and 2-year results have been reported previously (9, 12, 13). In brief, a total of 25 individuals that were planned for RYGB surgery were recruited between October 2011 and August 2012 from the Department of Endocrinology, Odense University Hospital and the Department of Endocrinology, Hospital South West Jutland, Denmark. Inclusion criteria were eligibility for RYGB according to Danish guidelines (age >25 years and BMI >50 kg/m^2^ or BMI >35 kg/m^2^ with at least one obesity-related comorbidity) and informed consent. Exclusion criteria for entering the study were peri-menopausal status, osteoporosis or bone metabolic disease or medications affecting bone metabolism. The 7-year follow up examinations were performed between October 2018 and June 2019. Supplements with calcium carbonate (1000–1200 mg daily) and vitamin D3 (38 mg daily) as well as a multivitamin tablet daily were advised post-surgery according to Danish national guidelines. Assessment of calcium and vitamin D intake and use of anti-osteoporotic treatments were performed using a questionnaire and chart review at the 7-year visit. The Regional Scientific Ethical Committee for Southern Denmark approved the study. All participants provided informed consent. 

### Biochemistry

At each visit a blood sample was obtained after an overnight fast. We measured parathyroid hormone (PTH) (Immunolite 2000, Siemens), follicle stimulation hormone (FSH), luteinizing hormone (LH) (both AutoDelfia, Perkin Elmer) and 25-hydroxy vitamin D (Cobas e11, Roche Diagnostics), and the coefficient of variation (CV) for these analyses ranged from 4 to 6% while measurements of procollagen type I amino-terminal propeptide (P1NP) and C-terminal telopeptide of type 1 collagen (CTX) (iSYS, Immunodiagnostic Systems, Tyne and Wear, UK) had CV’s ranging from 8 to 10%. CTX and P1NP from all visits were analysed in one batch.

### Dual energy x-ray absorptiometry (DEXA)

We measured areal BMD (aBMD) at the lumbar spine (L1–L4) and total hip region and whole body composition (lean and fat mass) using DEXA (Discovery, Hologic, Waltham, MA). Classification into normal BMD, osteopenia or osteoporosis was performed based on a T-score at the total hip or lumbar spine with intervals of >−1.0, ≤−1.0 to −2.4 or ≤−2.5, respectively. The references used for calculation of T-scores were provided by the manufacturer for the lumbar spine and the NHANES III for the total hip (14). Female reference values were applied in both women and men. Z-scores were calculated using reference data provided by the manufacturer. Quality control was performed as recommended by the manufacturer, including daily phantom scanning for x-ray tube constancy. The CV for lumbar spine and total hip BMD in our unit was 1.5% at both sites. 

### High resolution peripheral quantitative computed tomography (HR-pQCT)

Distal radius and distal tibia geometry, volumetric bone mineral density (vBMD), microarchitecture and estimated bone strength were measured using HR-pQCT (XtremeCT, Scanco Medical, Brütisellen, Switzerland). Standard evaluation software (Scanco Medical Software V6.0) was used for the evaluation of geometry, vBMD and trabecular microarchitecture (15). An extended cortical evaluation programme was applied for indices of cortical thickness and cortical porosity (16). A finite element analysis software (Finite Element Analysis Software v.1.15, Scanco Medical) was used for the estimation of bone strength (failure load). Up to three images at each skeletal site were obtained and post-acquisition grading of image quality and motion artefacts (1 being best and 5 being worst as recommended by the manufacturer) was performed by one author (SH), and images with adequate quality (≥3) were successfully obtained in all participants. Quality control was assessed using daily and weekly scan of phantoms (QRM, Möhrendorf, Germany). The CV’s were up to 0.8% for densities, 5.0% for trabecular microarchitecture indices, 7.2% for extended cortical indices and 1.7% for estimated failure load. 

### Statistical analyses

We report data as mean ± s.d. or median (range) depending on normal or non-normal distributions, respectively. A one way repeated-measures ANOVA was performed using baseline, 2- and 7-year values. In models where significant effects with time were found (as judged by the Huynh-Feldt corrected *P*-value to compensate for potential violations of sphericity), time-wise comparisons were performed to assess changes between baseline, 2 and 7 years. In cases where the dependent variable was not normally distributed, as judged by histograms or Shapiro–Wilk’s test, inverse, log or square root transformation was performed as appropriate. Spearman correlations were performed between changes in bone indices (baseline vs 7 years) and the following variables: baseline age, changes in body weight and lean mass and levels of bone remodelling markers at 7 years. *P*-values of 0.05 or below were considered significant. All statistical analyses were performed using Stata Statistical Software, release 12.0 (StataCorp LP).

## Results

### Clinical characteristics and biochemistry

**Figure 1:**
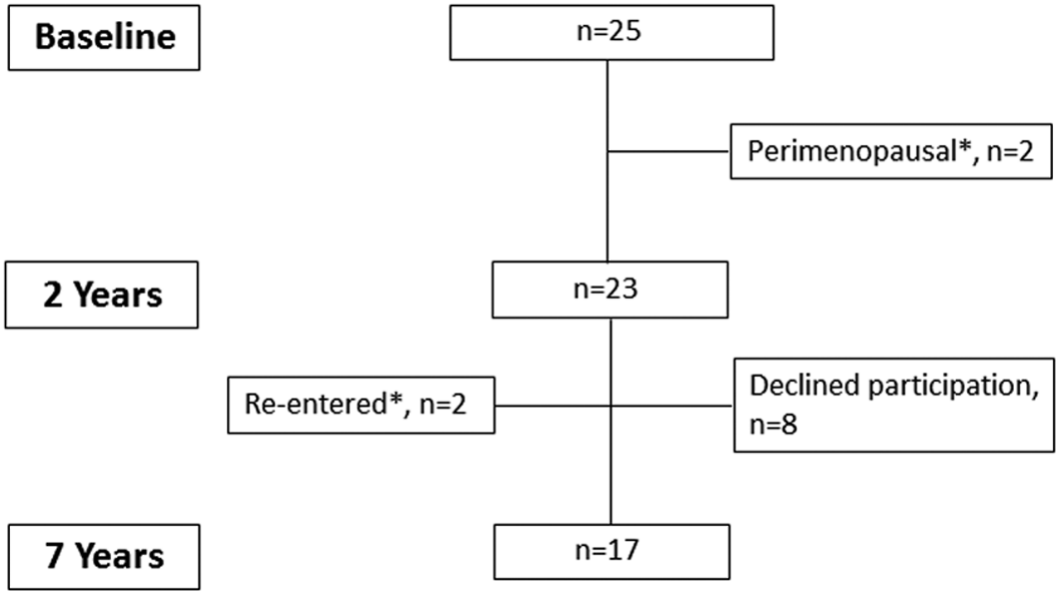
Diagram showing the number of participants at baseline, 2 and 7 years of follow-up. *Two women entered menopause within 2 years from surgery and were excluded from the previous 2-year analysis (9). In the present study, they were included in both the 2- and 7-year analyses.

The clinical characteristics of the 17 individuals that had RYGB performed and were examined at the 7-year follow-up are shown in Table 1. Of the 25 individuals that were examined at baseline, 17 (68%) were examined at the 7-years visit (Fig. 1). Age at baseline was 43 ± 9 years, and the cohort included seven men and ten women of whom nine were premenopausal and one was postmenopausal. From baseline to 2 years, two women entered menopause and additionally one woman entered menopause from year 2 to 7 with cessation of menses and rise in gonadotropins. As shown in Fig. 1 these two women had been excluded from the previous 1- and 2-year reports from this cohort (9, 12), although their results from baseline, 2 and 7 years are included in the current study. None of the participants used hormone replacement therapy. Ten participants reported daily intake of supplements with calcium (640 ± 207 mg daily) and vitamin D3 (24 ± 18 µg daily). One of the women that entered menopause during follow-up developed osteoporosis and received treatment with a bisphosphonate (four annual infusions of zoledronic acid 5 mg initiated 3 years after RYGB), and she was omitted from the data analyses of DEXA, HR-pQCT and bone remodelling markers.

**Table 1:** General characteristics and biochemistry results in patients at baseline and 2 and 7 years after Roux-en-y gastric bypass surgery.

	Baseline	2 years	7 years	% change baseline vs 7 years	% change 2 vs 7 years
*n*	17	–	–	–	–
Age (years)	43 ± 9	–	–	–	–
Sex (female/male)	10/7	–	–	–	–
Premenopausal*	9	7	6	–	–
Postmenopausal	1	–	4	–	–
Type 2 diabetes	5	–	–	–	–
Height (m)	174 ± 8	174 ± 8	174 ± 8	−0.1 ± 0.5	0.0 ± 0.4
Weight (kg)	127 ± 20	95 ± 19^‡^	101 ± 26	−21 ± 11^‡^	6 ± 12
Body mass index (kg/m^2^)	42 ± 6	31 ± 5^‡^	33 ± 8	−21 ± 11^‡^	5 ± 12
Lean mass (kg)	71 ± 16	62 ± 14^‡^	61 ± 15	−5 ± 7^‡^	−2 ± 6
Fat mass (kg)	55 ± 12	34 ± 11^‡^	41 ± 15	−27 ± 17^‡^	20 ± 24^§^
Ionized calcium (mmol/L)	1.24 ± 0.05	1.26 ± 0.04	1.23 ± 0.05	−0.7 ± 5	−2 ± 4
Parathyroid hormone (pmol/L)	5.5 ± 2.1	4.7 ± 2.3	5.9 ± 2.5	15 (−67 to 175)	16 (−57 to 80)
25-hydroxy-vitamin D (nmol/L)	38 ± 21	100 ± 27^‡^	100 ± 35	316 ± 385^‡^	−2 ± 30

Laboratory reference intervals: Ionized calcium (1.18–1.32 mmol/L), Parathyroid hormone (1.1–6.9 pmol/L).

*Note that three women became postmenopausal during follow-up. Shown as mean ± s.d. or median (range) as appropriate. † and ‡ indicate significance of differences compared to baseline. § and | indicate significance of differences between the 2- and 7-year values. ^‡^*P* < 0.001 and ^§^*P* < 0.05.

**Figure 2:**
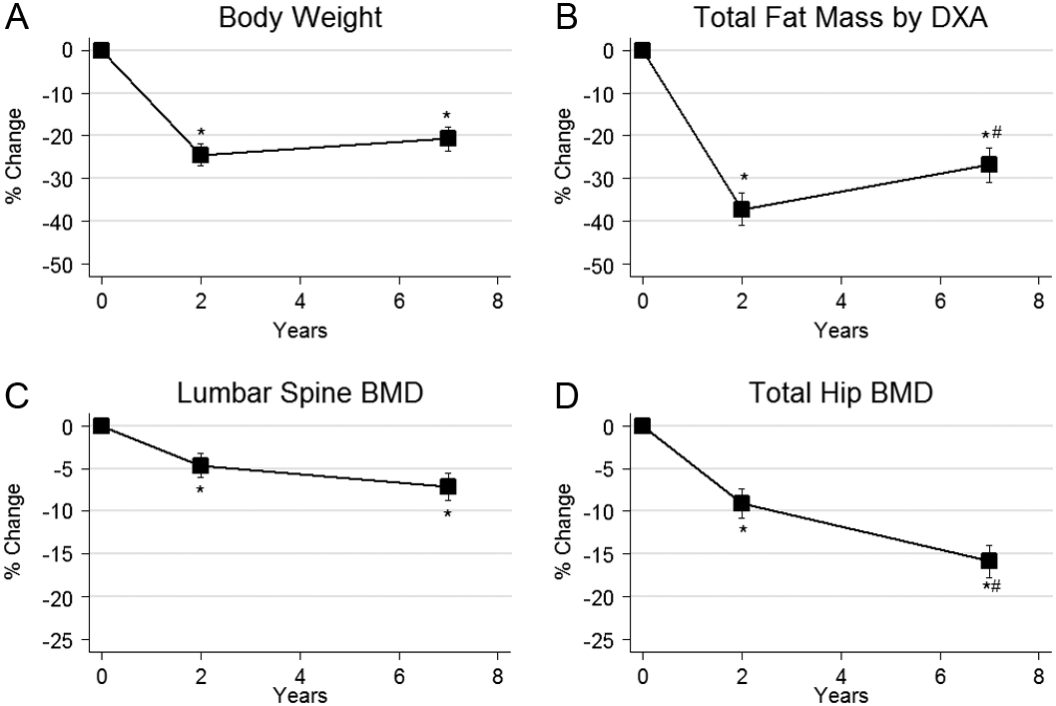
Mean changes in body weight (A), body fat mass (B), lumbar spine aBMD (C) and total hip aBMD by DEXA (D) after 2 and 7 years of follow-up. *P < 0.05 for comparison vs baseline and # for *P* < 0.05 vs 2-year value. Error bars = s.e.m.

**Figure 3:**
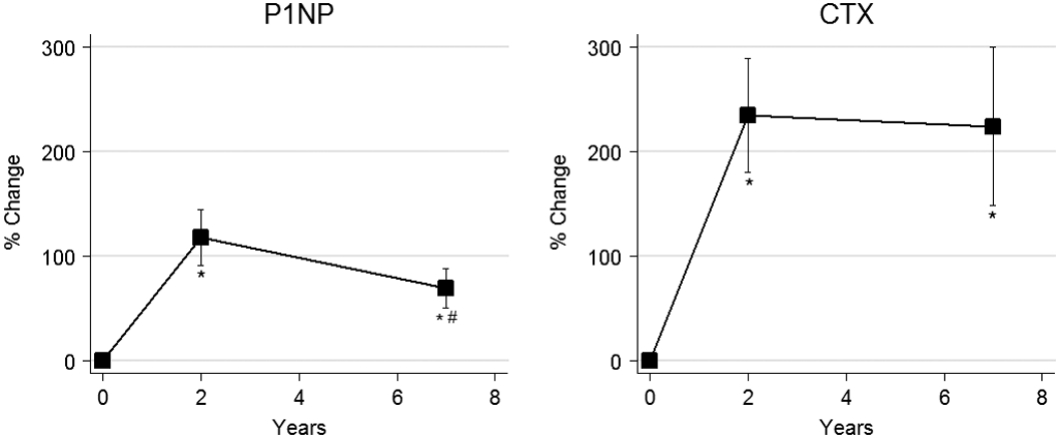
Mean changes in P1NP and CTX after 2 and 7 years of follow-up. *P < 0.001 for comparison vs baseline and # for *P* < 0.05 vs 2-year value. Error bars = s.e.m.

Before surgery participants had a mean weight of 127 ± 20 kg (corresponding to a BMI of 42 ± 6 kg/m^2^) and had a mean weight reduction of 31 ± 14 kg after 2 years (BMI 31 ± 5 kg/m^2^) and 27 ± 15 kg after 7 years (BMI 33 ± 8 kg/m^2^, *P* < 0.001 for all comparisons versus baseline). Corresponding declines in both lean mass and fat mass were observed (*P* < 0.001 for baseline comparisons). The minor rise in mean body weight between the 2- and 7-year visits was not significant (*P* = 0.15), but there was a significant increase in fat mass from 2 to 7 years (6 ± 9 kg, *P* < 0.05) (Fig. 2). There were no changes in ionized calcium or PTH during the study while 25-hydroxy vitamin D was markedly increased from baseline after 2 years and then plateaued (both *P* < 0.001). Ten had 25-hydroxy vitamin D below 50 nmol/L at baseline compared to two at 7 years. Blood samples for analyses of bone remodelling markers were available for 16, 16 and 14 individuals at baseline, 2- and 7-year visits, respectively. As shown in Fig. 3, compared to baseline, P1NP was increased after 2 years (117 ± 108%, *P* < 0.001) and then significantly declined towards 7 years (−17 ± 34%, *P* = 0.050 vs 2 years) although remaining above the baseline value (69 ± 71% *P* < 0.001). Similarly, CTX was increased after 2 years (235 ± 217%, *P* < 0.001) and remained elevated after 7 years (224 ± 284%, *P* < 0.001).

### Areal BMD by DEXA

DEXA results are shown in Fig. 2. Seven years after RYGB, lumbar spine BMD had declined by 8 ± 7% (*P* < 0.001) and total hip BMD by 17 ± 8% (*P* < 0.001) compared to baseline. Compared to the 2-year results, there was a continuous decline in total hip BMD (8 ± 7%, *P* < 0.001) while lumbar spine BMD was similar. 

After 7 years, three had developed osteoporosis, eight were osteopenic and six had normal BMD, while at baseline 15 had normal BMD and two had osteopenia. Z-scores at baseline, 2 and 7 years were 0.26 ± 1.19, −0.10 ± 1.34 and −0.17 ± 1.34 at the lumbar spine and 1.09 ± 0.95, 0.38 ± 1.21 and −0.05 ± 1.20 at the total hip, respectively.

### Bone geometry, vBMD, microarchitecture and estimated strength by HR-pQCT

In radius, a moderate decline in total vBMD was observed after 2 years with a loss of 14 ± 9% compared to baseline and 10 ± 7% compared to 2 years (both *P* < 0.001) (Table 2). A similar loss was found in trabecular vBMD (24 ± 12% vs baseline; 18 ± 14% vs 2 years, both *P* < 0.001), while the loss in cortical vBMD appeared of smaller magnitude (2 ± 3%, *P* < 0.001 vs baseline; 2 ± 2%, *P* < 0.05 vs 2 years). Changes were also observed in cortical microarchitecture with a reduction in cortical thickness (4 ± 7%, *P* < 0.05 vs baseline) and an increase in cortical porosity (33 ± 49%, *P* < 0.05 vs baseline). In the trabecular compartment, there were signs of ongoing deterioration of the trabecular network with reduction in trabecular number (13 ± 16%, *P* < 0.001 vs baseline), trabecular thickness (12 ± 8% vs baseline; 11 ± 10% vs 2 years, both *P* < 0.001) and an increase in trabecular separation (25 ± 30%, *P* < 0.001 vs baseline; 17 ± 34%, *P* < 0.05 vs 2 years) and trabecular network inhomogeneity (66 ± 94%, *P* < 0.001 vs baseline; 53 ± 116%, *P* < 0.05 vs 2 years). Overall, this led to a reduction in estimated bone strength of 15 ± 9% vs baseline and 11 ± 8% vs 2 years (both *P* < 0.001).

**Table 2:** Bone geometry, vBMD, microarchitecture and estimated bone strength using HR-pQCT in patients at baseline, 2 and 7 years after Roux-en-y gastric bypass surgery.

	Baseline (*n* = 16)	2 years (*n* = 16)	7 years (*n* = 16)	% change baseline vs 7 years	% change 2 vs 7 years
Radius					
Cortical area, mm^2^	71 ± 15	69 ± 16	66 ± 17	−6 ± 9^†^	−4 ± 8^‡^
Cortical thickness, mm	0.95 (0.54–1.12)	0.95 (0.48–1.09)	0.91 (0.50–1.10)	−4 ± 7^*^	−1 ± 7
Cortical porosity, %	1.30 (0.39–3.90)	1.41 (0.44–4.13)	1.98 (0.58–6.69)	33 ± 49^*^	13 ± 36
Trabecular area, mm^2^	266 ± 89	266 ± 88	268 ± 88	1 ± 3^†^	1 ± 2^‡^
Total vBMD, mg/cm^3^	339 ± 53	326 ± 54	294 ± 54	−13 ± 8^†^	−10 ± 7^§^
Cortical vBMD, mg/cm^3^	902 (768–987)	895 (734–971)	880 (721–932)	−2 ± 3^†^	−2 ± 2^‡^
Trabecular vBMD, mg/cm^3^	171 ± 48	160 ± 46^*^	131 ± 43	−24 ± 12^†^	−18 ± 14†
Trabecular number, 1/mm	2.12 ± 0.31	1.99 ± 0.29	1.85 ± 0.46	−13 ± 16^†^	−7 ± 19
Trabecular thickness, mm	0.067 ± 0.02	0.066 ± 0.01	0.058 ± 0.01	−12 ± 8^†^	−11 ± 10^§^
Trabecular separation, mm	0.395 (0.334–0.560)	0.423 (0.327–0.598)	0.437 (0.376–1.168)	25 ± 30^†^	17 ± 34^‡^
Trabecular network inhomogeneity, mm	0.151 (0.121–0.229)	0.174 (0.122–0.281)^*^	0.186 (0.140–0.818)	66 ± 94^†^	53 ± 116^‡^
Estimated failure load, *n*	5152 ± 1549	4922 ± 1484^*^	4272 ± 1400	−15 ± 9^†^	−11 ± 8^§^
Tibia					
Cortical area, mm^2^	160 ± 35	147 ± 32^†^	141 ± 37	−12 ± 8^†^	−4 ± 7
Cortical thickness, mm	1.38 ± 0.20	1.30 ± 0.19^†^	1.28 ± 0.21	−7 ± 8^†^	−2 ± 5
Cortical porosity, %	5.94 ± 2.68	6.05 ± 2.81^*^	7.06 ± 3.31	25 ± 44^†^	5 ± 17
Trabecular area, mm^2^	687 ± 132	697 ± 134^†^	702 ± 129	2 ± 2^†^	1 ± 1
Total vBMD, mg/cm^3^	324 ± 43	300 ± 53^†^	277 ± 48	−14 ± 8^†^	−7 ± 8^§^
Cortical vBMD, mg/cm^3^	883 ± 40	864 ± 53^†^	848 ± 55	−4 ± 4^†^	−2 ± 2^‡^
Trabecular vBMD, mg/cm^3^	190 ± 39	175 ± 42^†^	157 ± 35	−17 ± 10^†^	−9 ± 12^§^
Trabecular number, 1/mm	2.33 ± 0.33	2.11 ± 0.40^†^	2.06 ± 0.43	−12 ± 10^†^	−2 ± 13
Trabecular thickness, mm	0.066 (0.058–0.092)	0.067 (0.048–0.086)	0.064 (0.041–0.079)	−3 ± 14	−4 ± 11^‡^
Trabecular separation, mm	0.345 (0.287–0.512)	0.381 (0.320–0.709)^†^	0.411 (0.309–0.703)	19 ± 14^†^	6 ± 15
Trabecular network inhomogeneity, mm	0.140 (0.108–0.327)	0.157 (0.118–0.530)^†^	0.168 (0.113–0.489)	29 ± 26^†^	10 ± 24
Estimated failure load, *n*	13431 ± 2457	12631 ± 2695^†^	11341 ± 2627	−15 ± 8^†^	−8 ± 8^§^

Please note that one woman was omitted from the analyses due to bisphosphonate treatment. Data are means ± s.d. or median (range) as appropriate. * and † indicate significance of differences compared to baseline. ‡ and § indicate significance of differences between the 2- and 7-year values.

*P < 0.05, ^†^*P* < 0.001, ^‡^*P* < 0.05 and ^§^*P* < 0.001.

vBMD, volumetric bone mineral density.

In tibia, a pattern of change very similar to that in radius was observed. Total vBMD declined continuously (14 ± 8% vs baseline; 7 ± 8% vs 2 years, both *P* < 0.001) as did trabecular vBMD (17 ± 10% vs baseline;9 ± 12% vs 2 years, both *P* < 0.001) and cortical vBMD (4 ± 4%, *P* < 0.001 vs baseline; 2 ± 2% *P* < 0.05 vs 2 years). Thickness of the cortex was reduced (7 ± 8% vs baseline, *P* < 0.001), and cortical porosity was increased (25 ± 44%, *P* < 0.001 vs baseline). Evidence of worsening of the trabecular network was found with reduction compared to baseline in trabecular number (12 ± 10%, *P* < 0.001) and an increase in trabecular separation (19 ± 14%, *P* < 0.001) and trabecular network inhomogeneity (29 ± 26%, *P* < 0.001). Trabecular thickness was unchanged after 2 years but was reduced after 7 years (7 ± 11%, *P* < 0.05 vs 2 years). Estimated bone strength was reduced 15 ± 8% vs baseline and 8 ± 8% vs 2 years (both *P* < 0.001).

### Predictors of changes in bone indices

Baseline age was significantly associated with the decline in radius cortical vBMD after 7 years (*rho* = 0.60, *P* < 0.05) but not with changes in other HR-pQCT or DEXA outcomes. The reduction in body weight after 7 years was associated with declines in tibia cortical area (*rho* = 0.52, *P* < 0.05), tibia total vBMD (*rho* = 0.64, *P* < 0.01) and tibia trabecular vBMD (*rho* = 0.56, *P* < 0.05). The reduction in lean mass was also associated with declines in tibia cortical area (*rho* = 0.65, *P* < 0.01) and tibia total vBMD (*rho* = 0.58, *P* < 0.05) and additionally with reductions in tibia cortical thickness (*rho* = 0.58, *P* < 0.05) and trabecular number (*rho* = 0.50, *P* = 0.05) and with the increase in tibia trabecular area (*rho* = 0.58, *P* < 0.05). The level of P1NP and CTX at 7 years was associated with the decline in radius trabecular thickness (*rho’s* = 0.54 and 0.56 respectively, both *P* < 0.05) but not with other changes in HR-pQCT or DEXA indices. 

### Sensitivity analyses

A sensitivity analysis was performed excluding the two women who entered menopause during the study. Overall, findings were similar regarding DEXA, HR-pQCT and bone remodelling indices, although a few results became non-significant, namely declines in cortical porosity in radius and tibia from baseline to 7 years (*P* = 0.13 and *P* = 0.06, respectively) and declines from 2 to 7 years in radius in cortical area (*P* = 0.06), cortical vBMD (*P* = 0.07) and increase in trabecular area (*P* = 0.06). 

## Discussion

This is the first study to prospectively assess skeletal changes 7 years after RYGB. In accordance with our hypothesis, evidence of ongoing decline from year 2 to 7 in proximal femur BMD and deterioration in cortical and trabecular bone microarchitecture in the non weight-bearing radius and in the weight-bearing tibia were found causing substantial reductions in estimated bone strength. In spite of an unchanged body weight from 2 to 7 years post-surgery, most BMD and bone microarchitecture indices had further declined compared to the 2-year assessment, although generally with smaller magnitudes compared to the baseline to 2-year interval, suggesting a prolonged alteration of skeletal homeostasis with ongoing bone loss. 

In prospective studies, Raoof and colleagues (10) and Lindemann and colleagues (11) reported declines in lumbar spine and proximal femur areal BMD 5 years after RYGB. In agreement with our findings after 7 years, both studies observed an ongoing decline in proximal femur BMD from 2 to 5 years, while spine BMD did not significantly change in the latter study; a pattern of change similar to the one observed in our study. In the first mentioned study, supplements with calcium and vitamin D were not routinely advised, and a substantial proportion of participants developed secondary hyperparathyroidism that may explain the larger observed bone loss at the lumbar spine in this study. Also, a possible explanation for the lack of change in BMD at the lumbar spine is the increase in osteoarthritis at this site over time, which may falsely elevate BMD. Lindeman and colleagues (11) also studied changes in bone microarchitecture using HR-pQCT and similar to our findings, they reported declines in BMD, deterioration in radius and tibia microarchitecture and declines in estimated bone strength in both the radius and tibia. The magnitude of changes after 5 years in their study, as well as the pattern of change in cortical and trabecular microarchitecture, was overall very similar to the one we observed after 7 years, although for some indices their changes appear to supersede those found in our study. In particular, changes in cortical porosity seemed of larger magnitude in their study (269% in radius and 107% in tibia vs 33% and 25%, respectively in our study). Since image acquisition and analysis were similar in the two studies, differences are likely related to heterogeneity of the cohorts with their participants being on average 8 years older and with a larger proportion of female participants half of whom were postmenopausal at the time of surgery. This may be of importance, since postmenopausal women in particular have been shown to be at greater risk of bone loss after RYGB at least in the initial year after surgery (17). Also, in healthy adults, postmenopausal women have larger annual increases in cortical porosity compared to younger premenopausal women and men, and thus age-and sex related changes rather than effects of RYGB may explain this discrepancy (18, 19). 

The magnitude and pattern of change in bone microarchitecture across the lifespan in healthy individuals have been documented in previous studies using HR-pQCT (18, 20). While most bone microarchitecture indices are stable in young women and men, small annual changes (typically less than one percent) are observed from approximately mid-life and onwards. In postmenopausal women, where the largest changes occur, negative changes are primarily observed through cortical thinning and increasing cortical porosity with few changes in trabecular microarchitecture. Based on estimations from cross-sectional observations, these microarchitecture alterations lead to decreases in estimated bone strength (failure load) of 34% in radius and 26% in tibia in women from 35 to 80 years of age (20). Thus, the observed changes in patients after RYGB are substantial and the pattern of change very much resembles that observed with aging, although particularly the trabecular microarchitecture changes appear even more pronounced. In our study, after 7 years, failure load had declined on an average of 15% at both radius and tibia corresponding to approximately half of the expected relative change observed in healthy individuals until old age. Such declines in failure load may be of particular importance regarding actual fracture risk, since finite element estimated failure load using HR-pQCT have been shown to improve prediction of fracture beyond femoral neck BMD or estimations using the Fracture Risk Assessment Tool (FRAX) (21). 

According to BMD by DEXA, during the 7 years of follow-up, 88% of the cohort shifted from having normal BMD into having either osteopenia or osteoporosis. This occurred in spite of correction of vitamin D insufficiency in a number of participants and an overall substantial increase in 25-hydroxy vitamin D levels throughout follow-up which often cause a considerable increase in BMD. One woman was postmenopausal before surgery while three women entered menopause during study follow-up (of whom one was excluded from the data analysis due to treatment with zoledronic acid). Some of the observed changes may have been caused by a peri-menopausal bone loss rather than changes related to RYGB. In the sensitivity analyses, excluding the two women that entered menopause, the results were overall similar although for a few parameters changes became non-significant which may suggest that some of the observed changes were indeed related to menopause. 

Previous studies with 5 years of follow-up, including a study of patients with diabetes, have found persistent elevations in CTX 5 years after bariatric surgery indicating a prolonged increase in bone resorption (11, 22). Also, increases in P1NP after 5 years have been reported (11). In agreement with these studies, we found evidence of a sustained increase in bone remodelling with elevated levels of P1NP and CTX, where the magnitude of increase in CTX appeared more pronounced than the increase in P1NP. Thus, 7 years after surgery there were no signs of weaning of the negative effects of RYGB on skeletal homeostasis indicating a permanent imbalance leading to continuous bone loss. Accordingly, we found that the level of CTX after 7 years, as well as P1NP, were associated with the decline in trabecular thickness in radius although not with changes in other HR-pQCT or DEXA parameters. Also, we did observe a large increase in serum 25-hydroxy vitamin D after 2 and 7 years compared to baseline. This is in contrast to previous studies with a 5-year follow-up, where stable values compared to baseline were reported (10, 11). In our study, 59% of participants reported daily intake of calcium and vitamin D which likely explains the increase in 25-hydroxy vitamin D which may also mitigate secondary hyperparathyroidism potentially increasing bone remodelling. 

This study has some limitations. First, we had a small sample size and quite a large number of participants declined to participate in the 7-year visit and only 68% of the initial cohort was retained. This may have caused selection bias, in case those with the largest declines after 2 years were keener on participating compared to those with stable 2-year results causing an overestimation of effects. Participants were informed of their 2-year DEXA results and there were no evidence to suggest that those retained in the study had larger 2-year declines compared to those that were lost to follow-up. The limited number of participants also precluded firm assessment of predictors of bone changes or specific changes in subgroups such as women and men or pre- vs postmenopausal women. 

Second, a non-surgery control group was not available in this study and a direct comparison of age-related skeletal changes and changes induced by RYGB was not performed. 

This study also has some important strengths. Most importantly we had the longest so far follow-up that enabled documentation of longer-term adverse effects in the skeleton. The BMD in obese individuals is usually normal or high with favorable bone microarchitecture indices compared to normal weight individuals (13), and therefore with a few years of follow-up after RYGB, most individuals will likely still have a normal BMD and normal bone microarchitecture status. Results from this 7-year study highlight the need for continued assessment of bone health in individuals with prior RYGB. 

In conclusion, 7 years after RYGB, we found evidence of increased bone remodelling along with continuous declines in BMD and deteriorations of cortical and trabecular microarchitecture and declines in estimated bone strength compared to baseline and 2 years post-surgery results. These findings emphasize the need for regular assessment of bone health in post-bariatric surgery patients, and studies examining the potential use of anti-osteoporotic agents in relation to bariatric surgery are warranted.

## Declaration of interest

The authors declare that there is no conflict of interest that could be perceived as prejudicing the impartiality of this study.

## Funding

The study was supported by a grant from the Municipality Region of Southern Denmark.
